# Differentially expressed genes and canonical pathway expression in human atherosclerotic plaques – Tampere Vascular Study

**DOI:** 10.1038/srep41483

**Published:** 2017-01-27

**Authors:** Miska Sulkava, Emma Raitoharju, Mari Levula, Ilkka Seppälä, Leo-Pekka Lyytikäinen, Ari Mennander, Otso Järvinen, Rainer Zeitlin, Juha-Pekka Salenius, Thomas Illig, Norman Klopp, Nina Mononen, Reijo Laaksonen, Mika Kähönen, Niku Oksala, Terho Lehtimäki

**Affiliations:** 1 Department of Clinical Chemistry, Tampere University Hospital, Fimlab Laboratories and University of Tampere, School of Medicine, Finland; 2 Heart Center, Department of Cardio-Thoracic Surgery, Tampere University Hospital and University of Tampere, School of Medicine, Tampere, Finland; 3 Division of Vascular Surgery, Department of Surgery, Tampere University Hospital, Finland; 4 Research Unit of Molecular Epidemiology, Helmholtz Zentrum, German Research Center for Environmental Health, Munich, Germany; 5 Hannover Unified Biobank, Hannover Medical School, Hannover, Germany; 6 Institute for Human Genetics, Hannover Medical School, Hanover, Germany; 7 Department of Clinical Physiology, Tampere University Hospital and University of Tampere, School of Medicine, Finlan

## Abstract

Cardiovascular diseases due to atherosclerosis are the leading cause of death globally. We aimed to investigate the potentially altered gene and pathway expression in advanced peripheral atherosclerotic plaques in comparison to healthy control arteries. Gene expression analysis was performed (Illumina HumanHT-12 version 3 Expression BeadChip) for 68 advanced atherosclerotic plaques (15 aortic, 29 carotid and 24 femoral plaques) and 28 controls (left internal thoracic artery (LITA)) from Tampere Vascular Study. Dysregulation of individual genes was compared to healthy controls and between plaques from different arterial beds and Ingenuity pathway analysis was conducted on genes with a fold change (FC) > ±1.5 and false discovery rate (FDR) < 0.05. 787 genes were significantly differentially expressed in atherosclerotic plaques. The most up-regulated genes were osteopontin and multiple MMPs, and the most down-regulated were cell death-inducing DFFA-like effector C and A (CIDEC, CIDEA) and apolipoprotein D (FC > 20). 156 pathways were differentially expressed in atherosclerotic plaques, mostly inflammation-related, especially related with leukocyte trafficking and signaling. In artery specific plaque analysis 50.4% of canonical pathways and 41.2% GO terms differentially expressed were in common for all three arterial beds. Our results confirm the inflammatory nature of advanced atherosclerosis and show novel pathway differences between different arterial beds.

Atherosclerosis, the most common cause for cardiovascular diseases (CVDs) is a complex disease affecting millions of people around the world^1^. Genetic factors, environment, lifestyle choices and the various interactions between these affect the development of atherosclerotic plaques and modify individuals risk for adverse CVD events. Development of atherosclerotic plaque modifies the arterial wall through many metabolic pathways with inflammation and deposition of lipids being the most crucial processes involved. There is great variability in the development of this disease between individuals and even though atherosclerosis has a systemic nature, there are differences in gene-expression in plaques occurring in different arterial beds^2^. Also the prevalence of calcified or unstable plaques often varies according to vascular region^3^ and the on-going processes in a plaque differ greatly according to the stage of the plaque^4^.

Atherosclerosis begins with microscopic changes in the arterial wall. Accumulation of lipoproteins^5^ attracts inflammatory cells that begin to invade the intima^6^,^7^. As the disease progresses the arterial wall gathers more lipids and inflammatory cells (mainly T-cells and monocytes) and a visible fatty streak forms. Although the formation of a fatty streak is seen as a reversible event, it can be followed by the formation of fibrotic tissue leading to the stabilization of the plaque. The vascular smooth muscle cells begin to replicate contributing to the formation of the atheroma and the blood flow in the artery is impaired^7^,^8^. The plaque can also gather calcium and form a hard calcified plaque. The rupture or erosion of the the atherosclerotic plaque may result in a local thrombosis or release of distant thromboemboli, which both can have lethal consequences depending on the location of the plaque^7^,^9^.

Differentially expressed genes have been shown in various studies designed to specifically demonstrate the regulation of selected single genes in atherosclerosis. These studies have mostly been conducted *in vivo,* focusing on up-regulating or down-regulating selected genes, in order to find out their effect on the development and progression of atherosclerosis. Results show that changes in the expressions of target genes can also lead into the suppression of some atheroprotective qualities^10^,^11^. Previous studies have shown the inflammatory nature of atherosclerosis^12^, demonstrating the roles of different leukocytes present in atherosclerotic plaques^13^. Degradation and remodeling of the extracellular matrix^14^ and changes in the arterial wall^15^ are the most important processes related with atherosclerosis. Therefore, instead of single gene analysis to reveal the pathways – an unbiased whole genome wide pathway analyses against most recently updated gene-pathway databases are needed^16^,^17^,^18^. So far, a lot of data has been collected in murine and porcine models. Nevertheless, research on humans and human tissues is needed as genetically unaltered mice do not spontaneously develop atherosclerosis and the time frame and contributing risk factors differ greatly in animal models.

In ongoing Tampere Vascular Study (TVS)^2^ we aim to detect specific genes as well as pathways consisting of a set of genes differentially expressed in advanced atherosclerotic plaques compared to healthy non-atherosclerotic arteries (referred simply as differentially expressed). In our previous pilot work with 24 samples (6 controls and 18 cases)^2^ we established genes and pathways differentially expressed in atherosclerosis, including some MMPs and ApoC1 and upregulated proinflammatory pathways. In this study, with a substantially larger set of samples, and using novel Ingenuity pathway analysis (IPA) for the pathway analysis we aimed to further analyze the pathways and genes differentially expressed in peripheral advanced atherosclerotic plaques in humans. We also aimed to find possible differences between gene sets specific for plaques formed in certain arterial beds in different parts of arterial network. This larger sample set has been utilized in analyzing individual genes and their role in atherosclerotic plaques in hypothesis based studies^19^,^20^,^21^,^22^, and this study was performed to create a more comprehensive description of different expression of genes and pathways in atherosclerosis.

## Materials and Methods

### Tampere Vascular Study

The atherosclerotic vascular samples used in this study were obtained from patients subjected to carotid (N = 29) and femoral (N = 24) endarterectomy or an abdominal aortic bypass (N = 15) procedure (i.e., aortobifemoral bypass) due to occlusive atherosclerosis. The control vessel, left internal thoracic artery (LITA) (N = 28) samples were obtained during coronary artery bypass surgery^22^,^23^,^24^,^25^,^26^,^27^. Each sample was obtained from a different individual. All open vascular surgical procedures were performed at the Division of Vascular Surgery and the Heart Center at Tampere University Hospital between 2006 and 2009^27^. The study has been approved by the Ethics Committee of Pirkanmaa Hospital District and clinical investigation followed the principles of Helsinki declaration. Informed consent was acquired from patients enrolled in this study. In the present work the severity of atherosclerotic lesions in artery wall was histologically classified according to the American Heart Association classification (AHA classification I-VI)^28^. Demographics of the study population are presented in Supplementary File 1.

### RNA isolation and genome wide expression analysis

The fresh tissue samples were soaked in RNALater solution (Ambion Inc., Austin, TX, USA) and isolated with Trizol reagent (Invitrogen, Carlsbad, CA, USA) and the RNAEasy Kit (Qiagen, Valencia, CA, USA). The RNA quality as well as concentration was evaluated spectrophotometrically (BioPhotometer, Eppendorf, Wesseling-Berzdorf, Germany). The RNA isolation protocol was validated with Agilent RNA 6000 Nano Kit and the RNA integrity number (RIN) and the shape of the electropherogram were evaluated. In all the samples with successful gene expression profiling the 260/280 ratio was between 1.94–2.23. Over 23,000 known and candidate genes were analyzed with Illumina HumanHT-12 version 3 Expression BeadChip (Illumina Inc.), according to manufacturer’s instructions (Illumina, San Diego, CA, USA). Briefly, 200 ng aliquots of total RNA from each sample were amplified to cDNA using the Ambion’s Illumina RNA Amplification kit according to the instructions (Ambion, Inc., Austin, TX, USA). Samples of cRNA (1500 ng) were hybridized to Illumina’s Expression BeadChip arrays (Illumina). Hybridized biotinylated cRNA was detected with 1 μg/ml Cyanine3-streptavidine (Amersham Biosciences, Pistacataway, NJ, USA). BeadChips were scanned with the Illumina BeadArray Reader^2^. The accuracy of this array has been previously tested in our TVS validation study where the results of 192 differentially expressed genes were verified by RT-PCR^23^. The correlation of expression measurements between GWE and RT-PCR methods was good (r = 0.87, y = 0.151 + 0.586x), and the Bland-Altman plot showed that the FCs were in agreement with these two methods, although for highly up- or down-regulated transcripts, GWE yielding lower absolute FC values than in LDA^23^.

### Microarray array data analysis and multiple testing corrections

After background subtraction, raw intensity data were exported using the Illumina GenomeStudio software. Raw expression data were imported into R version 3.1.1 (http://www.r-project.org/), log2 transformed and normalized by the locally estimated scatterplot smoothing normalization method implemented in the R/Bioconductor package Lumi (www.bioconductor.org). Locally estimated scatterplot smoothing (LOESS) normalization for the data was selected because it gives the best accuracy in comparison with quantitative reverse transcription polymerase chain reaction data for artery samples^23^. Data quality control criteria included detection of outlier arrays based on the low number of robustly expressed genes and hierarchical clustering. Artery samples (n = 96: 68 plaque, 28 LITA) fulfilled all data quality control criteria, whilst 6 samples were discarded. To estimate fold changes between groups, we calculated differences between medians (in log2 scale) and then back transformed the log ratios to fold changes. To ease the interpretation, we replaced fold-change values that are <1 by the negative of its inverse. Statistical significance of differences in gene expression was assessed using the nonparametric Wilcoxon signed-rank test and the log-transformed data. Pathway analysis of the expression data was done with QIAGEN’s Ingenuity Pathway Analysis (IPA, QIAGEN Redwood City, www.qiagen.com/ingenuity) for genes with FC ± 1.5 and FDR < 0.05. Pathways with FDR < 0.05 (Benjamini-Hochberg method^29^) were found to be significantly differentially expressed. The GO-terms were analysed using GOrilla online tool^30^. The Venn diagrams were done using Venny 2.1.0 online tool^31^.

## Results

### Overall gene expression differences between atherosclerotic and atherosclerosis free arteries

787 of genes were significantly differentially expressed (FC > ±3.0, FDR < 0.05) in atherosclerotic plaques in comparison to healthy control arteries (Supplementary File 2). Table 1 lists the most significantly differentially expressed genes between all atherosclerotic plaque samples versus atherosclerosis free control arteries (LITAs) (All vs. LITA, up- or down-regulated, FC ≥ 15, FDR < 0.05) and the corresponding FC and p-values obtained from comparisons between different atherosclerotic plaque groups (aortic, carotid, femoral) and LITAs. The single most up-regulated gene was osteopontin (SPP1/secreted phophoprotein-1) (FC = 121.9 in all atherosclerotic plaques in comparison to controls). It was highly up-regulated in all analyzed atherosclerotic arterial beds (FC > 100) and it was the most differentially expressed gene in femoral and carotid plaques (Supplementary Files 1 and 2).

Many of the most up-regulated genes are previously known inflammatory genes, such as CD4, CD28 and chemokines and chemokine receptor genes. Other significantly up-regulated gene families are the MMPs (MMP7, MMP9 and MMP12, FC > 28 for all when comparing all atherosclerotic plaques to controls, Table 1). In addition, several gene families associated with lipid transport were the most differentially expressed ones in atherosclerotic plaques (Table 1, Supplementary File 2). Apolipoprotein-D (ApoD) was down-regulated in all the studied arterial beds, whilst ApoE and ApoC1 were both significantly up-regulated in atherosclerotic plaques. ApoC1 was especially up-regulated in carotid plaques (FC = 53.2). In addition, cell death-inducing DFFA-like effector C and A (CIDEC, CIDEA) were significantly down-regulated in atherosclerotic plaques in comparison to control LITA. CIDEC and CIDEA were down-regulated in the plaques from all the studied arterial beds but there were large differences in the magnitude of the down-regulation (Table 1, Supplementary File 2).

### Gene expression differences between plaques derived from different arterial beds

We also found significant differences in the gene expression patterns between plaques taken from different types of arteries from different vascular regions. Most significant differences were seen between plaques from aortic and carotid arteries (FC > 5, FDR < 0.05) (Table 2). Especially ApoD and chemokine ligand 14, CXCL14, genes were down-regulated when comparing aortic to carotid artery plaque. Twenty-two genes were differentially expressed when comparing plaques from femoral artery to those from aorta whilst the plaques from carotid and femoral artery presented the most similar gene expression patterns (Table 2). The most differentially expressed genes between different arterial plaques (FC > 5.0, FDR < 0.05) are listed in Table 2 and more completely in Supplementary File 3.

### Altered canonical pathways in advanced atherosclerotic plaques in comparison to LITA

**Figure 1:**
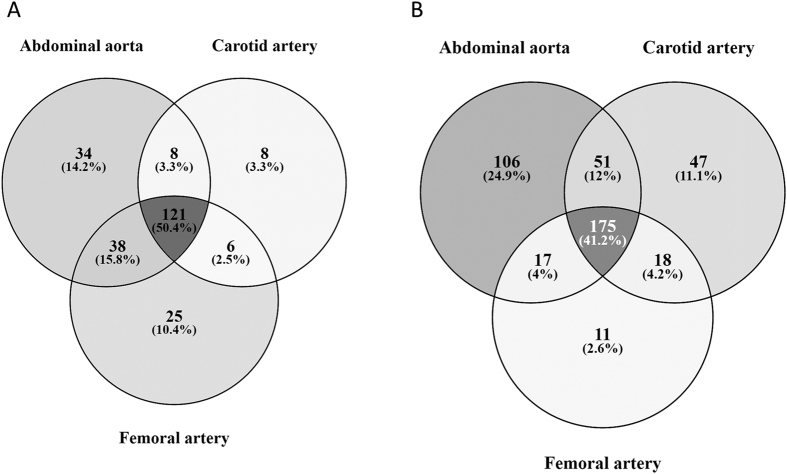
A Venn diagram showing the number of (**A**) Ingenuity Canonical Pathways and (**B**) GO-terms differentially expressed in different arterial beds and the overlapping of those pathways/GO-terms between atherosclerotic plaques from different arterial beds.

Ingenuity pathway analysis identified 156 altered pathways between atherosclerotic plaques and healthy arteries, with FDR < 0.05, when analyzing genes with FC > ±1.5. In aortic, carotid and femoral plaques 201, 143 and 190 pathways were respectively differentially expressed, of which 121 were shared by all of the arterial beds (Fig. 1A). The top 10 canonical pathways of each arterial bed in comparison to the healthy control to, as well as the top 25 pathways from the analysis of all atherosclerotic arteries versus healthy controls are shown in Tables 3 and 4 and the full list of pathways with significant differential expression is provided in Supplementary File 4. The number of genes up- and downregulated is shown as well as the ratio of differentially expressed genes on the pathway to all the genes on the pathway. Most of the pathways that showed significant (FDR < 0.05) up-regulation in atherosclerotic plaques were related to the inflammatory process in the arterial wall, including inflammatory markers, such as CD4 and CD28, as well as CXCR4. The pathways that are differentially expressed are all closely related to the inflammation and ensuing apoptosis of the cells of the arterial wall. The 121 pathways differentially expressed in all the atherosclerotic arteries are mostly related to the leukocyte activity of arterial wall, involving for example pathways that lead to adhesion and diapedesis of agranulocytes. Abdominal aortic plaques were found to have more unique differentially expressed pathways, including up-regulation of several distinct inflammatory pathways, whilst the 25 pathways differentially expressed only in femoral plaques included several down-regulated catabolic pathways (Table 4).

GO-term analyses showed that 288 Go terms were up-regulated in atherosclerotic plaques in comparison to controls (Supplementary File 5). Similarly to IPA pathway analysis gene sets up-regulated in individual arterial beds greatly overlapped (Fig. 1B) and were mostly related to inflammatory processes (Supplementary File 4). As with the IPA pathways, aortic plaques had the greatest number of unique differentially expressed gene sets. Only GO term “ncRNA metabolic process” was down-regulated in carotid plaques in comparison to controls.

## Discussion

Our results underline the importance of osteopontin in the development of atherosclerosis and suggest that the different expression of cell death-inducing DFFA-like effector C and A may also be involved in the pathogenesis of atherosclerosis. These results offer more specified and wider information on the biochemical pathways differentially expressed (against most recently updated data bases) in atherosclerotic plaques, artery type specific expression information and further extend and specify the previously published results. We demonstrate the extensively individual genes that are differentially expressed and biological pathways in atherosclerotic plaques using the unique sample collection of the TVS. Perisic *et al*.^32^ conducted a similar study, which yielded similar results, where atherosclerotic plaques were compared to blood samples from, we consider using a healthy artery to be a more valid method as the tissues are more alike. The differentially expressed genes and pathways reported here are mostly involved in the inflammatory events previously assumed to occur in the atherosclerotic arterial wall, i.e. the recruitment and translocation of leucocytes through the intimal layer and infiltration of the vessel wall, differentiation of macrophages to foam cells, and the remodeling of extracellular matrix, which affects the stability of the plaque.

Osteopontin was the most up-regulated gene in the atherosclerotic plaques in comparison to healthy control arteries. Ikeda *et al*. have previously shown atherosclerotic severity dependent expression of osteopontin in human aortic plaques^33^ and osteopontin has also been connected to coronary artery disease in a large GWAS study^34^. Osteopontin is a key component in inflammatory processes, involved in cell migration and as a chemoattractant for monocytes^35^ and in adhesion in vascular and T-cells^35^. Our results showing the up-regulation of osteopontin are supported also by previous evidence that it is expressed by foam cells derived from smooth muscle cells^33^ and that it has been indicated in the calcification process of the atherosclerotic plaque. Osteopontin has the ability to bind calcium^25^,^36^ which is presumed to be a response to prevent further mineralization of the plaque^37^. Osteopontin also participates in the recruitment and further differentiation of macrophages^36^, through this role, the expression levels of osteopontin also correlate with the plaques’ stability as the amount and subtype of macrophages correlate with plaque stability. Due to the wide range of effects and excessive expression in the plaque tissue, osteopontin is clearly one of the key players in the pathogenesis of atherosclerosis. Interferon beta has been shown to modulate the expression of osteopontin by Chen *et al*.^38^ in multiple sclerosis and our results suggest it might be an interesting pharmacological approach in atherosclerosis as well.

Also cathepsins, a group of proteinases including some collagenases and bone reabsorption associated enzymes, some of which are known to participate in vascular diseases^39^ were significantly up-regulated. Now our results give context and show the role of cathepsins (CTS) in relation to other differentially expressed genes as part of the complex process of atherosclerosis pathogenesis.

Several multiple matrix metalloproteinases (MMPs) showed very substantial up-regulation in atherosclerotic plaques. Our results further affirm the role of two MMPs (MMP12 and −9) previously associated with stroke in GWAS analysis by the METASTROKE collaboration^40^, and carotid artery atherosclerosis^32^. MMPs are peptidases that have been shown to participate in vascular remodeling^14^ and plaque formation^14^ as well as in plaque rupture^39^ in atherosclerotic arteries. They also have modulatory functions in cell signaling^41^. In our pathway analysis MMPs were found in canonical pathways related to adhesion and diapedesis of leukocytes, facilitating the transmigration of leukocytes through endothelial layer. Of the MMP family MMP7, MMP9, MMP11 and MMP12 had the highest up-regulation levels with different proportional levels in different arterial beds. The abundance of MMP9 in femoral plaques and MMP12 in carotid plaques supports the idea of MMPs expression in plaque formation being site-, and possibly sex-, specific^42^,^43^.

Differentially expressed lipid metabolism related genes included CIDEC, CIDEA and apolipoproteins E, D and C1. The up-regulation of ApoE and ApoC1 in our advanced atherosclerotic artery samples seem to support the correlations that have been found with lipid deposits and cholesterol accumulation^44^ and plaque size^45^. Interestingly, in our study we also showed that ApoD was down-regulated in the plaque tissue. CIDEC and CIDEA have previously been associated with the intake of lipids and formation of lipid droplets in macrophage derived foam cells^46^. Therefore, their down-regulation may indicate decreased foam cell formation in advanced atherosclerotic plaques or possibly other currently unknown roles in the atherosclerotic plaque^47^.

Several chemokines, as well as their receptors, were up-regulated, the most important ones being CCL5 and CCL2. These have been shown to be involved in chemokinesis, dendritic cell signaling^48^, monocyte recruitment^49^ and enhancement of vascular permeability, all of which fit the pathology of atherosclerosis and have been associated with several pathological conditions^50^, including, but not limited to, atherosclerosis^50^.

From our results we can speculate that the genes differently expressed in plaques from different arterial beds might help to explain as to why certain plaques tend to be more stable and calcify or become fibrotic. The delay to surgery is shorter in carotid surgery, as opposed to femoral artery or abdominal aorta, because they often contain an unstable plaque and are highly symptomatic. This is likely to explain a part of the different expression patterns in the carotid plaques. As the carotid plaques are more frequently unstable the role of MMP12 and −7 might warrant further research, as our results show that of all the arterial beds these MMPs have the greatest expression in carotid artery samples. Also, our results suggest the role of homeobox-genes as stabilizing elements because two homeobox genes, HOXC6 and HOXA5 (Table 2), were the significantly down-regulated genes in carotid artery as opposed to the femoral artery and abdominal aorta.

Our pathway analysis results are well in line with previous analysis done with smaller sample sets or with those deduced from GWAS results. When comparing IPA pathways enriched with genes associated with CAD^34^ or large artery stroke^51^ in GWAS, our data indicated 44 pathways out of the 96 pathways connected with CAD were differentially expressed and 11 pathways out of the 16 connected with large artery stroke^17^. Moreover 12 out of the 16 pathways previously connected to large artery stroke were differentially expressed in carotid plaques in comparison to controls^17^. The GO terms with significant different expression are also very similar to those found by Nai *et al*.^16^. Although their analysis contained only carotid plaques and they used different control material - the healthy arteries obtained near the plaques -the pathways identified in their study well match our results. In addition, 9 of the 10 most up-regulated GO terms in their study are up-regulated also in our data, and although “cytokine production” is not up-regulated in our results, “regulation of cytokine production” is ref. 16. Similar GO terms have also been shown to be enriched in the study conducted by Perisic *et al*.^32^.

Our pathway analysis highlights the importance of inflammation related pathways also in advanced atherosclerotic plaques. The pathways activated show that the movement and attachment of leukocytes is a crucial part of the pathogenesis of atherosclerosis. Also the remodeling of extracellular matrix and differentiation of cells producing extracellular components were activated, such as the pathway of hepatic stellate cell activation. The GO term search further affirmed the results of the inflammatory nature of atherosclerosis, as many of the GO terms were inflammation related. Our results also show that similar pathways are differentially expressed in peripheral atherosclerotic plaques from different locations, but abdominal aortic plaques have more inflammatory pathways up-regulated and down-regulation of several catabolic pathways can only be seen in femoral plaques.

### Limitations of the study

The different hemodynamics associated with plaque localization on the arterial wall might have contributed to the results, along with the fact that controls had coronary artery disease, which implies similar systemic factors. In our study we used the LITA samples as control, as LITA is usually quite impervious to atherosclerosis^52^, and is therefore widely used as a control material in similar studies. Control samples from corresponding arterial beds free of atherosclerosis and from healthy subjects without systemic risk factors could have provided more precise analysis. However, due to ethical reasons we were not able to obtain such samples. In endarterectomy, intima mainly containing the plaque and inner media are removed. In non-atherosclerotic LITA control arteries, it is not feasible to remove the adventitia and outer media. Due to this setting, the LITA samples include the thin adventitia consisting mainly of loose connective tissue, whilst plaque samples lack the adventitial layer. This should not reduce the validity or hamper interpretation of our results. The suitability of LITAs as controls^16^ is further supported by our results being well in line with the Gene ontology results performed with carotid plaques and adjacent healthy tissues as controls by Perisic *et al*.^32^. In addition, we have previously shown that the tissue morphology is similar in LITA samples, healthy carotid artery, and abdominal aorta and verified the similar expression patterns of focal adhesion molecules in these healthy arterial beds^53^.

Both cases and controls had similar prevalence of statin users, with 87.5% of cases and 73.5% of controls (Supplementary File 1) and we therefore consider the groups comparable. High rate of statin usage may have some effect on gene expression but does not explain the differences between cases and controls. However, it is noteworthy that we cannot separate the changes in gene expression caused by the pathological state from those caused by statins or other medication. As majority of the samples were advanced atherosclerotic plaques, more research is needed to determine the dominant genes and pathways active in more early phases of the pathogenesis of atherosclerosis. We do not use qRT-PCR to further affirm our results as it has been shown by Raitoharju *et al*.^23^ that when FC > 3 the specificity is 100% and when FC > 1.5 it is 73.7%.

In conclusion, the development of atherosclerotic plaque extensively disturbs the function of the human arterial wall and leads to different expression of a multitude of genes and pathways. Our gene expression analysis from human peripheral atherosclerotic plaques confirm the previous associations of many differentially expressed gene families, such as matrix metalloproteinases, apolipoproteins and cholesterol related receptors. Also, the role of osteopontin as one of the central genes in atherosclerosis was underlined in our results, as it was the single gene with the greatest fold change in our samples, possibly having a role in the calcification process and progression of atherosclerotic plaques. We also report for the first time the down-regulation of ApoD in human atherosclerotic plaques, and although its association with atherosclerosis is indisputable its role is still not completely understood. The inflammatory nature of the disease was further supported here, as many inflammatory pathways were differentially expressed in the advanced peripheral atherosclerotic plaques. As seen in Fig. 1, atherosclerosis has significant differences in gene expression depending on the site of the arterial plaque, and only 50.4% of IPA pathways and 41.2% of GO terms are in common between all atherosclerosis affected arteries. These results will serve as a good map for further research on the genes and pathways behind human atherosclerosis.

## Additional Information

**How to cite this article**: Sulkava, M. *et al*. Differentially expressed genes and canonical pathway expression in human atherosclerotic plaques – Tampere Vascular Study. *Sci. Rep.* **7**, 41483; doi: 10.1038/srep41483 (2017).

**Publisher's note:** Springer Nature remains neutral with regard to jurisdictional claims in published maps and institutional affiliations.

## Supplementary Material

Supplementary Information

Supplementary File 2

Supplementary File 3

Supplementary File 4

Supplementary File 5

## Figures and Tables

**Table 1:** The most significantly differentially expressed genes in All atherosclerotic plaques versus atherosclerosis free control arteries (LITA), (FC ≥ ± 15 and FDR < 0.05) and the corresponding FC and p-values from comparisons between atherosclerotic plaques from different arterial beds (abdominal aorta, carotid, femoral) compared to LITA.

Gene symbol	ACCESSION	All vs. LITA		Aorta vs. LITA		Carotid vs. LITA		Femoralis vs LITA	
		FC	P-value	FC	p-value	FC	p-value	FC	p-value
SPP1	NM_001040058.1	121.9	1.82*10^−13^	104.3	2.73*10^−8^	135.7	1.19*10^−15^	105.4	3.16*10^−14^
SPP1	NM_000582.2	109.1	1.82*10^−13^	90.9	2.73*10^−8^	113.9	1.19*10^−15^	99.9	3.16*10^−14^
MMP12	NM_002426.2	88.9	1.82*10^−13^	72.4	2.73*10^−8^	124.9	1.19*10^−15^	68.3	3.16*10^−14^
MMP9	NM_004994.2	76.1	1.82*10^−13^	32.0	2.73*10^−8^	98.8	1.19*10^−15^	76.9	3.16*10^−14^
CIDEC	NM_022094.2	−61.2	2.91*10^−11^	−17.0	2.00*10^−3^	−90.4	1.19*10^−15^	−50.1	3.71*10^−10^
KIAA1881	XM_001130790.1	−60.1	9.97*10^−12^	−17.7	2.73*10^−8^	−80.1	1.19*10^−15^	−50.9	6.60*10^−11^
LOC642113	XM_936253.1	53.3	5.44*10^−12^	135.0	2.91*10^−8^	36.1	8.72*10^−12^	38.7	3.71*10^−10^
MMP7	NM_002423.3	52.5	2.21*10^−13^	17.8	3.93*10^−8^	91.8	1.19*10^−15^	36.2	3.16*10^−14^
LOC647450	XM_936518.1	52.4	5.12*10^−12^	153.5	2.91*10^−8^	47.6	8.72*10^−12^	32.5	2.94*10^−10^
LOC652493	XM_941953.1	52.1	3.55*10^−12^	127.7	2.91*10^−8^	45.2	4.17*10^−12^	32.1	1.82*10^−10^
LOC652694	XM_942302.1	47.2	3.14*10^−12^	129.4	2.14*10^−8^	46.0	2.48*10^−12^	22.2	1.43*10^−10^
MMP12	NM_002426.2	47.1	7.00*10^−13^	72.4	2.73*10^−8^	63.2	1.19*10^−15^	25.5	1.43*10^−10^
APOD	NM_001647.2	−39.7	1.24*10^−10^	−1.4	3.76*10^−2^	−68.1	1.19*10^−15^	−43.9	3.16*10^−14^
APOC1	NM_001645.3	36.2	2.21*10^−13^	30.3	9.94*10^−11^	53.2	2.38*10^−15^	32.8	6.33*10^−14^
PLA2G7	NM_005084.2	29.9	2.21*10^−13^	12.5	5.97*10^−10^	43.6	1.19*10^−15^	28.0	3.16*10^−14^
MMP7	NM_002423.3	28.3	2.21*10^−13^	17.8	1.99*10^−10^	54.6	1.19*10^−15^	21.2	3.16*10^−14^
KIAA1199	NM_018689.1	25.5	1.82*10^−13^	31.0	4.97*10^−11^	26.5	1.19*10^−15^	19.5	3.16*10^−14^
CIDEA	NM_001279.2	−24.5	6.22*10^−11^	−17.9	5.42*10^−5^	−30.4	8.13*10^−13^	−23.4	4.46*10^−8^
ADAMDEC1	NM_014479.2	24.2	1.82*10^−13^	26.1	4.97*10^−11^	28.5	1.19*10^−15^	15.0	3.16*10^−14^
SCARA5	NM_173833.4	−22.8	6.16*10^−13^	−3.3	1.08*10^−6^	−27.0	1.19*10^−15^	−23.8	3.16*10^−14^
ACP5	NM_001611.2	22.6	4.78*10^−13^	7.4	3.40*10^−8^	31.4	1.19*10^−15^	32.0	3.16*10^−14^
IGLL1	NM_020070.2	20.0	7.83*10^−12^	38.1	1.99*10^−10^	19.2	1.39*10^−11^	16.0	1.70*10^−9^
LOC652102	XM_941434.1	19.0	5.23*10^−11^	58.9	4.97*10^−11^	16.6	2.53*10^−10^	11.9	7.40*10^−8^
APOE	NM_000041.2	17.6	7.45*10^−13^	15.8	1.34*10^−7^	27.7	2.38*10^−15^	16.4	3.16*10^−14^
PLIN	NM_002666.3	−17.3	5.87*10^−11^	−12.9	4.66*10^−5^	−19.0	1.44*10^−12^	−18.5	1.70*10^−9^
ACTA1	NM_001100.3	−16.3	7.40*10^−11^	−9.3	7.02*10^−7^	−19.6	2.26*10^−14^	−9.2	3.86*10^−7^
RGS1	NM_002922.3	15.8	6.57*10^−13^	23.9	4.97*10^−11^	12.0	8.13*10^−13^	12.6	3.80*10^−13^
OLR1	NM_002543.3	15.8	3.70*10^−13^	20.6	5.97*10^−10^	15.4	5.35*10^−14^	17.1	1.27*10^−13^
MYOC	NM_000261.1	−15.2	3.05*10^−13^	−11.1	2.24*10^−9^	−17.8	1.19*10^−15^	−10.9	6.33*10^−14^

^a^Abbreviations: FC = fold changes (FC); FDR = False discovery rate; LITA = left internal thoracic artery.

**Table 2:** The most significantly differentially expressed (FC ≥ ± 5, FDR < 0.05) genes between atherosclerotic plaques from plaques from different arterial beds (abdominal aorta, carotid, femoral).

SYMBOL	ACCESSION	FC	P	FDR	SYMBOL	ACCESSION	FC	P	FDR
**Aorta vs carotid**					**Carotid vs femoral**				
APOD	NM_001647.2	43.4	2.87*10^−7^	7.15*10^−5^	HOXC6	NM_004503.3	−17.5	2.57*10^−15^	3.41*10^−11^
CXCL14	NM_004887.3	22.3	1.04*10^−10^	2.15*10^−7^		AK123741	−8.9	2.57*10^−15^	3.41*10^−11^
HOXC6	NM_004503.3	16.2	8.70*10^−12^	8.98*10^−8^	MMP13	NM_002427.2	−7.5	3.77*10^−9^	3.33*10^−6^
PLA2G2A	NM_000300.2	16.1	4.42*10^−9^	3.26*10^−6^	HOXC8	NM_022658.3	−6.7	2.57*10^−15^	3.41*10^−11^
PI16	NM_153370.2	14.2	7.95*10^−9^	5.02*10^−6^	LMO3	NM_018640.3	6.5	1.13*10^−9^	1.16*10^−6^
SOST	NM_025237.2	−10.5	3.52*10^−7^	8.23*10^−5^	HOXA5	NM_019102.2	−6.2	2.57*10^−15^	3.41*10^−11^
IL6	NM_000600.1	9.7	1.65*10^−10^	2.74*10^−7^	PITX1	NM_002653.3	−6.0	4.88*10^−14^	1.89*10^−10^
RASD1	NM_016084.3	9.0	7.95*10^−9^	5.02*10^−6^	HOXC4	NM_014620.4	−5.8	2.57*10^−15^	3.41*10^−11^
LOC653879	XM_936226.1	8.6	7.66*10^−7^	1.46*10^−4^	HOXA9	NM_152739.3	−5.7	2.57*10^−15^	3.41*10^−11^
THBS4	NM_003248.3	8.6	2.61*10^−10^	3.62*10^−7^	SFRP1	NM_003012.3	5.2	1.13*10^−5^	1.09*10^−3^
HOXA5	NM_019102.2	8.4	8.70*10^−12^	8.98*10^−8^	**Femoral vs aorta**				
C3	NM_000064.1	7.3	1.53*10^−7^	4.80*10^−5^	APOD	NM_001647.2	−27.8	2.85*10^−5^	9.15*10^−3^
DARC	NM_002036.2	7.2	1.33*10^−6^	2.19*10^−4^	CXCL14	NM_004887.3	−16.8	6.96*10^−6^	4.56*10^−3^
SCARA5	NM_173833.4	6.5	1.11*10^−6^	1.93*10^−4^	PI16	NM_153370.2	−12.7	3.53*10^−7^	9.36*10^−4^
C4orf7	NM_152997.2	6.4	1.01*10^−4^	5.34*10^−3^	PLA2G2A	NM_000300.2	−11.5	3.91*10^−6^	3.46*10^−3^
APOLD1	NM_030817.1	6.2	8.70*10^−12^	8.98*10^−8^	SFRP1	NM_003012.3	−11.5	5.77*10^−6^	4.22*10^−3^
SLC14A1	NM_015865.2	−6.0	2.26*10^−6^	3.30*10^−4^	IL6	NM_000600.1	−8.3	1.01*10^−5^	5.47*10^−3^
CD19	NM_001770.4	5.8	8.32*10^−6^	8.88*10^−4^	LOC653879	XM_936226.1	−8.3	2.13*10^−6^	2.64*10^−3^
CD79A	NM_021601.3	5.8	5.19*10^−6^	6.14*10^−4^	SPON1	NM_006108.2	−7.3	3.95*10^−5^	1.04*10^−2^
C6	NM_000065.1	5.8	6.09*10^−11^	1.62*10^−7^	C4orf7	NM_152997.2	−6.9	1.15*10^−4^	1.75*10^−2^
CCL19	NM_006274.2	5.5	6.22*10^−8^	2.34*10^−5^	CCL21	NM_002989.2	−6.8	2.62*10^−6^	2.83*10^−3^
NLF2	XM_940314.2	5.5	3.24*10^−9^	2.76E-*10^−6^	CCL19	NM_006274.2	−6.3	1.65*10^−7^	8.06*10^−4^
LOC651751	XM_940969.1	5.5	2.26*10^−6^	3.30*10^−4^	FCRLA	NM_032738.3	−6.3	3.21*10^−6^	3.07*10^−3^
FBLN1	NM_001996.2	5.5	6.22*10^−8^	2.34*10^−5^	GREM1	NM_013372.5	−6.2	1.11*10^−8^	1.71*10^−4^
NR4A2	NM_006186.2	5.3	1.21*10^−9^	1.34*10^−6^	C3	NM_000064.1	−6.1	3.21*10^−6^	3.07*10^−3^
SPON1	NM_006108.2	5.3	1.90*10^−6^	2.89*10^−4^	CD79A	NM_001783.3	−5.9	4.76*10^−6^	3.88*10^−3^
GREM1	NM_013372.5	5.3	3.24*10^−9^	2.76*10^−6^	SCARA5	NM_173833.4	−5.8	5.84*10^−4^	3.75*10^−2^
C7	NM_000587.2	5.3	6.09*10^−11^	1.62*10^−7^	LOC652694	XM_942302.1	−5.8	1.21*10^−5^	5.83*10^−3^
SVEP1	NM_153366.2	5.2	3.52*10^−7^	8.23*10^−5^	CD79A	NM_021601.3	−5.5	1.21*10^−5^	5.83*10^−3^
FOSB	NM_006732.1	5.2	2.37*10^−9^	2.27*10^−6^	DARC	NM_002036.2	−5.4	5.84*10^−4^	3.75*10^−2^
LOC401845	XM_377426.1	5.0	1.76*10^−5^	1.55*10^−3^	CD19	NM_001770.4	−5.4	3.36*10^−5^	9.77*10^−3^
					LOC651751	XM_940969.1	−5.3	1.44*10^−5^	6.28*10^−3^
					ABCA8	NM_007168.2	−5.0	6.96*10^−6^	4.56*10^−3^

^a^Abbreviations: FC = fold changes (FC); FDR = False discovery rate.

**Table 3:** Top 25 pathways differentially expressed in advanced atherosclerotic plaques when compared to the healthy left internal thoracic artery (LITA).

Ingenuity Canonical Pathways	B-H p-value (FDR)^a^	Ratio: differentially expressed/all pathway genes	Downregulated genes on the pathway	Up-regulated genes on the pathway	Genes not differentially expressed on the pathway
Antigen Presentation Pathway	3.89*10^−10^	0.730	0/37 (0%)	27/37 (73%)	10/37 (27%)
Hepatic Fibrosis/Hepatic Stellate Cell Activation	7.41*10^−10^	0.404	21/183 (11%)	53/183 (29%)	109/183 (60%)
Dendritic Cell Maturation	7.41*10^−10^	0.407	14/177 (8%)	58/177 (33%)	105/177 (59%)
Crosstalk between Dendritic Cells and Natural Killer Cells	7.08*10^−8^	0.472	6/89 (7%)	36/89 (40%)	47/89 (53%)
Leukocyte Extravasation Signaling	7.08*10^−8^	0.369	23/198 (12%)	50/198 (25%)	125/198 (63%)
Role of Macrophages. Fibroblasts and Endothelial Cells in Rheumatoid Arthritis	7.94*10^−8^	0.331	29/296 (10%)	69/296 (23%)	198/296 (67%)
Granulocyte Adhesion and Diapedesis	1.95*10^−7^	0.373	9/177 (5%)	57/177 (32%)	111/177 (63%)
Type I Diabetes Mellitus Signaling	7.24*10^−7^	0.418	3/110 (3%)	43/110 (39%)	64/110 (58%)
Axonal Guidance Signaling	4.17*10^−6^	0.288	56/434 (13%)	69/434 (16%)	309/434 (71%)
TREM1 Signaling	4.47*10^−6^	0.453	1/75 (1%)	33/75 (44%)	41/75 (55%)
LXR/RXR Activation	5.62*10^−6^	0.388	16/121 (13%)	31/121 (26%)	74/121 (61%)
Agranulocyte Adhesion and Diapedesis	1.29*10^−5^	0.339	17/189 (9%)	47/189 (25%)	125/189 (66%)
Communication between Innate and Adaptive Immune Cells	1.35*10^−5^	0.416	2/89 (2%)	35/89 (39%)	52/89 (58%)
Role of Pattern Recognition Receptors in Recognition of Bacteria and Viruses	1.35*10^−5^	0.376	6/125 (5%)	41/125 (33%)	78/125 (62%)
CD28 Signaling in T Helper Cells	1.35*10^−5^	0.381	7/118 (6%)	38/118 (32%)	73/118 (62%)
Role of NFAT in Regulation of the Immune Response	1.35*10^−5^	0.345	14/171 (8%)	45/171 (26%)	112/171 (65%)
LPS/IL-1 Mediated Inhibition of RXR Function	2.09*10^−5^	0.321	29/221 (13%)	42/221 (19%)	150/221 (68%)
Graft-versus-Host Disease Signaling	2.19*10^−5^	0.500	1/48 (2%)	23/48 (48%)	24/48 (50%)
CTLA4 Signaling in Cytotoxic T Lymphocytes	7.08*10^−5^	0.398	6/88 (7%)	29/88 (33%)	53/88 (60%)
NF-Î°B Signaling	7.08*10^−5^	0.331	16/172 (9%)	41/172 (24%)	115/172 (67%)
Production of Nitric Oxide and Reactive Oxygen Species in Macrophages	7.08*10^−5^	0.328	16/180 (9%)	43/180 (24%)	121/180 (67%)
ILK Signaling	8.13*10^−5^	0.324	35/185 (19%)	25/185 (14%)	125/185 (68%)
FcÎ^3^ Receptor-mediated Phagocytosis in Macrophages and Monocytes	8.91*10^−5^	0.387	7/93 (8%)	29/93 (31%)	57/93 (61%)
Integrin Signaling	1.02*10^−4^	0.314	30/207 (14%)	35/207 (17%)	142/207 (69%)
Complement System	1.38*10^−4^	0.514	4/37 (11%)	15/37 (41%)	18/37 (49%)

^a^p-value calculated with Benjamini-Hochberg method.

**Table 4:** Top 10 differentially expressed canonical pathways from atherosclerotic plaques from different arterial beds (abdominal aorta, carotid, femoral) in comparison to left internal thoracic artery controls.

Ingenuity Canonical Pathways	B-H p-value (FDR)	Ratio: differentially expressed/all pathway genes	Downregulated genes on the pathway	Up-regulated genes on the pathway	Genes not differentially expressed on the pathway
**Aortic vs LITA**					
Hepatic Fibrosis/Hepatic Stellate Cell Activation	5.01*10^−13^	0.470	23/183 (13%)	63/183 (34%)	97/183 (53%)
Dendritic Cell Maturation	1.00*10^−12^	0.469	15/177 (8%)	68/177 (38%)	94/177 (53%)
Role of Macrophages. Fibroblasts and Endothelial Cells in Rheumatoid Arthritis	3.89*10^−10^	0.382	32/296 (11%)	81/296 (27%)	183/296 (62%)
Granulocyte Adhesion and Diapedesis	1.74*10^−9^	0.429	8/177 (5%)	68/177 (38%)	101/177 (57%)
Crosstalk between Dendritic Cells and Natural Killer Cells	2.00*10^−9^	0.528	6/89 (7%)	41/89 (46%)	42/89 (47%)
Leukocyte Extravasation Signaling	1.32*10^−8^	0.404	21/198 (11%)	59/198 (30%)	118/198 (60%)
Axonal Guidance Signaling	1.66*10^−8^	0.334	64/434 (15%)	81/434 (19%)	289/434 (67%)
Type I Diabetes Mellitus Signaling	2.29*10^−8^	0.473	3/110 (3%)	49/110 (45%)	58/110 (53%)
Antigen Presentation Pathway	5.62*10^−8^	0.676	0/37 (0%)	25/37 (68%)	12/37 (32%)
TREM1 Signaling	9.33*10^−8^	0.520	1/75 (1%)	38/75 (51%)	36/75 (48%)
**Carotid vs LITA**					
Hepatic Fibrosis/Hepatic Stellate Cell Activation	3.31*10^−10^	0.432	25/183 (14%)	54/183 (30%)	104/183 (57%)
Dendritic Cell Maturation	5.75*10^−9^	0.418	16/177 (9%)	58/177 (33%)	103/177 (58%)
Axonal Guidance Signaling	1.86*10^−7^	0.320	61/434 (14%)	78/434 (18%)	295/434 (68%)
Granulocyte Adhesion and Diapedesis	1.86*10^−7^	0.395	11/177 (6%)	59/177 (33%)	107/177 (60%)
Crosstalk between Dendritic Cells and Natural Killer Cells	1.86*10^−7^	0.483	6/89 (7%)	37/89 (42%)	46/89 (52%)
Antigen Presentation Pathway	3.16*10^−7^	0.649	0/37 (0%)	24/37 (65%)	13/37 (35%)
Leukocyte Extravasation Signaling	6.76*10^−7^	0.374	23/198 (12%)	51/198 (26%)	124/198 (63%)
TREM1 Signaling	7.59*10^−7^	0.493	1/75 (1%)	36/75 (48%)	38/75 (51%)
Agranulocyte Adhesion and Diapedesis	4.90*10^−6^	0.365	19/189 (10%)	50/189 (26%)	120/189 (63%)
Role of Macrophages. Fibroblasts and Endothelial Cells in Rheumatoid Arthritis	6.03*10^−6^	0.328	29/296 (10%)	68/296 (23%)	199/296 (67%)
**Femoral vs LITA**					
Leukocyte Extravasation Signaling	3.98*10^−11^	0.404	26/198 (13%)	54/198 (27%)	118/198 (60%)
Hepatic Fibrosis/Hepatic Stellate Cell Activation	1.48*10^−10^	0.404	20/183 (11%)	54/183 (30%)	109/183 (60%)
Dendritic Cell Maturation	1.48*10^−10^	0.407	15/177 (8%)	57/177 (32%)	105/177 (59%)
Antigen Presentation Pathway	4.57*10^−8^	0.649	0/37 (0%)	24/37 (65%)	13/37 (35%)
Role of Macrophages. Fibroblasts and Endothelial Cells in Rheumatoid Arthritis	8.13*10^−8^	0.324	28/296 (9%)	68/296 (23%)	200/296 (68%)
Granulocyte Adhesion and Diapedesis	1.58*10^−7^	0.367	7/177 (4%)	58/177 (33%)	112/177 (63%)
TREM1 Signaling	1.62*10^−7^	0.48	1/75 (1%)	35/75 (47%)	39/75 (52%)
Crosstalk between Dendritic Cells and Natural Killer Cells	2.88*10^−6^	0.427	6/89 (7%)	32/89 (36%)	51/89 (57%)
Integrin Signaling	3.24*10^−6^	0.333	33/207 (16%)	36/207 (17%)	138/207 (67%)
Production of Nitric Oxide and Reactive Oxygen Species in Macrophages	3.55*10^−6^	0.344	19/180 (11%)	43/180 (24%)	118/180 (66%)

## References

[b1] Gargiulo P. . Ischemic heart disease in systemic inflammatory diseases. An appraisal. Int. J. Cardiol. 170, 286–290 (2014). 24331863 10.1016/j.ijcard.2013.11.048

[b2] Levula M. . Genes involved in systemic and arterial bed dependent atherosclerosis–Tampere Vascular study. PLoS One 7, e33787 (2012). 22509262 10.1371/journal.pone.0033787 PMC3324479

[b3] Herisson F. . Carotid and femoral atherosclerotic plaques show different morphology. Atherosclerosis 216, 348–354 (2011). 21367420 10.1016/j.atherosclerosis.2011.02.004

[b4] Conde L. . Novel associations for coronary artery disease derived from genome wide association studies are not associated with increased carotid intima-media thickness, suggesting they do not act via early atherosclerosis or vessel remodeling. Atherosclerosis 219, 684–689 (2011). 21924425 10.1016/j.atherosclerosis.2011.08.031

[b5] Williams K. J. & Tabas I. The response-to-retention hypothesis of early atherogenesis. Arterioscler. Thromb. Vasc. Biol. 15, 551–561 (1995). 7749869 10.1161/01.atv.15.5.551 PMC2924812

[b6] Stary H. C. Changes in components and structure of atherosclerotic lesions developing from childhood to middle age in coronary arteries. Basic Res. Cardiol. 89 Suppl 1, 17–32 (1994). 7945171 10.1007/978-3-642-85660-0_2

[b7] Ross R. Atherosclerosis–an inflammatory disease. N. Engl. J. Med. 340, 115–126 (1999). 9887164 10.1056/NEJM199901143400207

[b8] Bentzon J. F., Otsuka F., Virmani R. & Falk E. Mechanisms of plaque formation and rupture. Circ. Res. 114, 1852–1866 (2014). 24902970 10.1161/CIRCRESAHA.114.302721

[b9] Starke R. M. . Vascular smooth muscle cells in cerebral aneurysm pathogenesis. Transl. Stroke Res. 5, 338–346 (2014). 24323713 10.1007/s12975-013-0290-1

[b10] Gimbrone M. A. Jr. & Garcia-Cardena G. Vascular endothelium, hemodynamics, and the pathobiology of atherosclerosis. Cardiovasc. Pathol. 22, 9–15 (2013). 22818581 10.1016/j.carpath.2012.06.006 PMC4564111

[b11] Leonarduzzi G., Gamba P., Gargiulo S., Biasi F. & Poli G. Inflammation-related gene expression by lipid oxidation-derived products in the progression of atherosclerosis. Free Radic. Biol. Med. 52, 19–34 (2012). 22037514 10.1016/j.freeradbiomed.2011.09.031

[b12] Tuttolomondo A. . Atherosclerosis as an inflammatory disease. Curr. Pharm. Des. 18, 4266–4288 (2012). 22390643 10.2174/138161212802481237

[b13] Tuomisto T. T. . Gene expression in macrophage-rich inflammatory cell infiltrates in human atherosclerotic lesions as studied by laser microdissection and DNA array: overexpression of HMG-CoA reductase, colony stimulating factor receptors, CD11A/CD18 integrins, and interleukin receptors. Arterioscler. Thromb. Vasc. Biol. 23, 2235–2240 (2003). 14576072 10.1161/01.ATV.0000102551.91154.96

[b14] Galis Z. S. & Khatri J. J. Matrix metalloproteinases in vascular remodeling and atherogenesis: the good, the bad, and the ugly. Circ. Res. 90, 251–262 (2002). 11861412

[b15] Zhou J., Li Y. S. & Chien S. Shear stress-initiated signaling and its regulation of endothelial function. Arterioscler. Thromb. Vasc. Biol. 34, 2191–2198 (2014). 24876354 10.1161/ATVBAHA.114.303422 PMC4169328

[b16] Nai W. . Identification of novel genes and pathways in carotid atheroma using integrated bioinformatic methods. Sci. Rep. 6, 18764 (2016). 26742467 10.1038/srep18764 PMC4705461

[b17] Pasterkamp G. . Human Validation of Genes Associated With a Murine Atherosclerotic Phenotype. Arterioscler. Thromb. Vasc. Biol (2016). 10.1161/ATVBAHA.115.306958 27079880

[b18] Ramsey S. A. . Epigenome-guided analysis of the transcriptome of plaque macrophages during atherosclerosis regression reveals activation of the Wnt signaling pathway. PLoS Genet. 10, e1004828 (2014). 25474352 10.1371/journal.pgen.1004828 PMC4256277

[b19] Oksala N. . Kindlin 3 (FERMT3) is associated with unstable atherosclerotic plaques, anti-inflammatory type II macrophages and upregulation of beta-2 integrins in all major arterial beds. Atherosclerosis 242, 145–154 (2015). 26188538 10.1016/j.atherosclerosis.2015.06.058

[b20] Oksala N. . Association of neuroimmune guidance cue netrin-1 and its chemorepulsive receptor UNC5B with atherosclerotic plaque expression signatures and stability in human(s): Tampere Vascular Study (TVS). Circ. Cardiovasc. Genet. 6, 579–587 (2013). 24122613 10.1161/CIRCGENETICS.113.000141

[b21] Fan Y. M. . Upstream Transcription Factor 1 (USF1) allelic variants regulate lipoprotein metabolism in women and USF1 expression in atherosclerotic plaque. Sci. Rep. 4, 4650 (2014). 24722012 10.1038/srep04650 PMC3983598

[b22] Turpeinen H. . Proprotein convertases in human atherosclerotic plaques: the overexpression of FURIN and its substrate cytokines BAFF and APRIL. Atherosclerosis 219, 799–806 (2011). 21889147 10.1016/j.atherosclerosis.2011.08.011

[b23] Raitoharju E. . A comparison of the accuracy of Illumina HumanHT-12 v3 Expression BeadChip and TaqMan qRT-PCR gene expression results in patient samples from the Tampere Vascular Study. Atherosclerosis 226, 149–152 (2013). 23177970 10.1016/j.atherosclerosis.2012.10.078

[b24] Oksala N. . ADAM-9, ADAM-15, and ADAM-17 are upregulated in macrophages in advanced human atherosclerotic plaques in aorta and carotid and femoral arteries–Tampere vascular study. Ann. Med. 41, 279–290 (2009). 19253070 10.1080/07853890802649738

[b25] Oksala N. . Carbonic anhydrases II and XII are up-regulated in osteoclast-like cells in advanced human atherosclerotic plaques-Tampere Vascular Study. Ann. Med. 42, 360–370 (2010). 20509747 10.3109/07853890.2010.486408

[b26] Turpeinen H. . A genome-wide expression quantitative trait loci analysis of proprotein convertase subtilisin/kexin enzymes identifies a novel regulatory gene variant for FURIN expression and blood pressure. Hum. Genet. 134, 627–636 (2015). 25813623 10.1007/s00439-015-1546-5

[b27] Raitoharju E. . miR-21, miR-210, miR-34a, and miR-146a/b are up-regulated in human atherosclerotic plaques in the Tampere Vascular Study. Atherosclerosis 219, 211–217 (2011). 21820659 10.1016/j.atherosclerosis.2011.07.020

[b28] Stary H. C. . A definition of advanced types of atherosclerotic lesions and a histological classification of atherosclerosis. A report from the Committee on Vascular Lesions of the Council on Arteriosclerosis, American Heart Association. Circulation 92, 1355–1374 (1995). 7648691 10.1161/01.cir.92.5.1355

[b29] Benjamini Y. & Hochberg Y. Controlling the False Discovery Rate: A Practical and Powerful Approach to Multiple Testing. *Journal of the Royal Statistical Society*. Series B (Methodological) 57, 289–300 (1995).

[b30] Eden E., Navon R., Steinfeld I., Lipson D. & Yakhini Z. GOrilla: a tool for discovery and visualization of enriched GO terms in ranked gene lists. BMC Bioinformatics 10, 48–2105–10–48 (2009). 19192299 10.1186/1471-2105-10-48 PMC2644678

[b31] http://bioinfogp.cnb.csic.es/tools/venny/index.html.

[b32] Perisic L. . Gene expression signatures, pathways and networks in carotid atherosclerosis. J. Intern. Med. 279, 293–308 (2016). 26620734 10.1111/joim.12448

[b33] Ikeda T., Shirasawa T., Esaki Y., Yoshiki S. & Hirokawa K. Osteopontin mRNA is expressed by smooth muscle-derived foam cells in human atherosclerotic lesions of the aorta. J. Clin. Invest. 92, 2814–2820 (1993). 8254036 10.1172/JCI116901 PMC288482

[b34] Schunkert H. . Large-scale association analysis identifies 13 new susceptibility loci for coronary artery disease. Nat. Genet. 43, 333–338 (2011). 21378990 10.1038/ng.784 PMC3119261

[b35] Lund S. A., Giachelli C. M. & Scatena M. The role of osteopontin in inflammatory processes. J. Cell. Commun. Signal. 3, 311–322 (2009). 19798593 10.1007/s12079-009-0068-0 PMC2778587

[b36] O’Regan A. & Berman J. S. Osteopontin: a key cytokine in cell-mediated and granulomatous inflammation. Int. J. Exp. Pathol. 81, 373–390 (2000). 11298186 10.1046/j.1365-2613.2000.00163.x PMC2517746

[b37] Wolak T. Osteopontin - a multi-modal marker and mediator in atherosclerotic vascular disease. Atherosclerosis 236, 327–337 (2014). 25128758 10.1016/j.atherosclerosis.2014.07.004

[b38] Chen M. . Regulatory effects of IFN-beta on production of osteopontin and IL-17 by CD4+ T Cells in MS. Eur. J. Immunol. 39, 2525–2536 (2009). 19670379 10.1002/eji.200838879

[b39] Newby A. C. Proteinases and plaque rupture: unblocking the road to translation. Curr. Opin. Lipidol. 25, 358–366 (2014). 25089553 10.1097/MOL.0000000000000111

[b40] Traylor M. . A novel MMP12 locus is associated with large artery atherosclerotic stroke using a genome-wide age-at-onset informed approach. PLoS Genet. 10, e1004469 (2014). 25078452 10.1371/journal.pgen.1004469 PMC4117446

[b41] Fanjul-Fernandez M., Folgueras A. R., Cabrera S. & Lopez-Otin C. Matrix metalloproteinases: evolution, gene regulation and functional analysis in mouse models. Biochim. Biophys. Acta 1803, 3–19 (2010). 19631700 10.1016/j.bbamcr.2009.07.004

[b42] Serra R. . The role of matrix metalloproteinases and neutrophil gelatinase-associated lipocalin in central and peripheral arterial aneurysms. Surgery 157, 155–162 (2015). 25444221 10.1016/j.surg.2014.06.008

[b43] Zhou W., Chai H., Ding R. & Lam H. Y. Distribution of inflammatory mediators in carotid and femoral plaques. J. Am. Coll. Surg. 211, 92–98 (2010). 20610254 10.1016/j.jamcollsurg.2010.02.054

[b44] Martins I. J. . Apolipoprotein E, cholesterol metabolism, diabetes, and the convergence of risk factors for Alzheimer’s disease and cardiovascular disease. Mol. Psychiatry 11, 721–736 (2006). 16786033 10.1038/sj.mp.4001854

[b45] Noto A. T., Mathiesen E. B., Brox J., Bjorkegren J. & Hansen J. B. The ApoC-I content of VLDL particles is associated with plaque size in persons with carotid atherosclerosis. Lipids 43, 673–679 (2008). 18509687 10.1007/s11745-008-3193-2

[b46] Wang J. . Identification by microarray technology of key genes involved in the progression of carotid atherosclerotic plaque. Genes Genet. Syst. 89, 253–258 (2014). 25948119 10.1266/ggs.89.253

[b47] Xu L., Zhou L. & Li P. CIDE proteins and lipid metabolism. Arterioscler. Thromb. Vasc. Biol. 32, 1094–1098 (2012). 22517368 10.1161/ATVBAHA.111.241489

[b48] Niessner A. & Weyand C. M. Dendritic cells in atherosclerotic disease. Clin. Immunol. 134, 25–32 (2010). 19520615 10.1016/j.clim.2009.05.006 PMC2821659

[b49] Leuschner F. . Therapeutic siRNA silencing in inflammatory monocytes in mice. Nat. Biotechnol. 29, 1005–1010 (2011). 21983520 10.1038/nbt.1989 PMC3212614

[b50] O’Connor T., Borsig L. & Heikenwalder M. CCL2-CCR2 Signaling in Disease Pathogenesis. *Endocr Metab*. Immune Disord. Drug Targets 15, 105–118 (2015). 10.2174/1871530315666150316120920 25772168

[b51] Traylor M. . Genetic risk factors for ischaemic stroke and its subtypes (the METASTROKE collaboration): a meta-analysis of genome-wide association studies. Lancet Neurol. 11, 951–962 (2012). 23041239 10.1016/S1474-4422(12)70234-X PMC3490334

[b52] Sajja L. R. & Mannam G. Internal thoracic artery: anatomical and biological characteristics revisited. Asian Cardiovasc. Thorac. Ann. 23, 88–99 (2015). 24585304 10.1177/0218492314523629

[b53] von Essen M. . Talin and vinculin are downregulated in atherosclerotic plaque; Tampere Vascular Study. Atherosclerosis 255, 43–53 (2016). 27816808 10.1016/j.atherosclerosis.2016.10.031

