# Supplementary material for: Differentially expressed genes and canonical pathway expression in human atherosclerotic plaques – Tampere Vascular Study
Source: Sci Rep. 2017 Jan 27;7:41483. doi: 10.1038/srep41483 (PMC5270243; doi:10.1038/srep41483)
Supplement: Supplementary Information [file srep41483-s1.doc]

**Differentially expressed genes and canonical pathway expression in human atherosclerotic plaques – Tampere Vascular Study**

Miska Sulkava^1^, BM; Emma Raitoharju^1^, PhD; Mari Levula^1^, PhD; Ilkka Seppälä^1^, MSc, BM; Leo-Pekka Lyytikäinen^1^, MD; Ari Mennander^2^, MD, PhD; Otso Järvinen^2^, MD, PhD; Rainer Zeitlin^3^, MD, PhD; Juha-Pekka Salenius^3^, MD, PhD; Thomas Illig^4, 5, 6^, MD, PhD; Norman Klopp^4, 5^, PhD; Nina Mononen^1^, PhD; Reijo Laaksonen^1^, MD, PhD; Mika Kähönen^7^, MD, PhD; Niku Oksala^1,3^, MD, PhD, DSc; Terho Lehtimäki^1^, MD, PhD.

**Supplementary materials list:**

Supplementary file 1. Demographics of the study population and a box-plot showing the expression of SPP1 over different arterial beds.

Supplementary file 2. Dysregulated genes in atherosclerotic arteries in comparison to healthy left internal thoracic artery (LITA) controls.

Supplementary file 3.The genes differentially expressed in different arterial beds with each other.

Supplementary file 4. The IPA analysis results.

Supplementary file 5. The GO term analysis.

**Supplementary file 1**. Demographics of the study population and a box-plot showing the expression of SPP1 over different arterial beds.

Demographics of the study population.

|  | | **Controls** | **Cases** | |
| --- | --- | --- | --- | --- |
| Number of subjects | 28 | | | 68 |
| Age. years (mean. SD) | 69.0 (8.3) | | | 70.0 (10.4) |
| Men (%) | 87.5 | | | 67.6 |
| Body mass index. kg/m^2^ (median. SD) | 28.2 (5.3) | | | 26.0 (4.0) |
| History of smoking (%) | 66.7 | | | 75.0 |
| Diabetes (%) | 29.2 | | | 23.5 |
| Hypercholesterolemia (%) | 91.7 | | | 67.6 |
| Hypertension (%) | 100.0 | | | 82.4 |
| Statin user (%) | 87.5 | | | 73.5 |
| Blood pressure medication (%) | 96.3 | | | 80.9 |
| Diabetes medication (for the diabetics of the group, %) | 88.9 | | | 76.5 |
| Coronary artery disease (%) | 100.0 | | | 29.4 |
| Myocardial infarction (%) | 33.3 | | | 13.2 |

Box-plot showing the expression of osteopontin (SPP1) over all the atherosclerotic arterial beds (carotid artery, abdominal aorta and femoral artery) with the control artery (LITA) on the left.


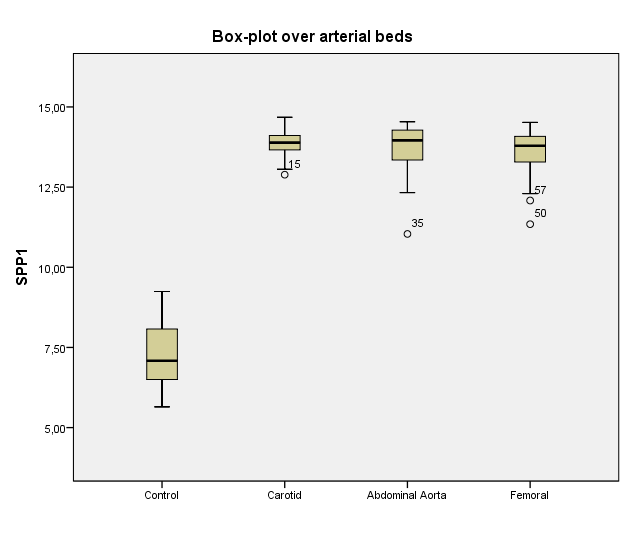


SPP1 accession NM_001040058.1
